# Strategies for financing social health insurance schemes for providing universal health care: a comparative analysis of five countries

**DOI:** 10.1080/16549716.2020.1868054

**Published:** 2021-01-20

**Authors:** Ama P. Fenny, Robert Yates, Rachel Thompson

**Affiliations:** a Institute of Statistical, Social and Economic Research (ISSER), University of Ghana, Accra, Ghana; b Centre for Global Health Security, Chatham House, The Royal Institute of International Affairs, London, UK

**Keywords:** Health financing, Kenya, Ghana, Ethiopia, Rwanda, Tanzania

## Abstract

**Background**: Universal Health Coverage has become a political priority for many African countries yet there are clear challenges in achieving this goal. Though social health insurance is considered a mechanism for providing financial protection, less well documented in the literature is evidence from countries in Africa who are at various stages of adopting this financing strategy as a way to improve health insurance coverage for their populations.

**Objectives**: The study investigates whether social health insurance schemes are effectively and efficiently covering all groups. The objective is to provide evidence of how these schemes have been implemented and whether the fundamental goals are met. The selected countries are Ghana, Rwanda, Tanzania, Kenya and Ethiopia. The study draws lessons from the literature about how policy tools can be used to reduce financial barriers whilst ensuring a broad geographic coverage in Africa.

**Methods**: The study relies primarily on a review of literature, both documented and grey matter, which include key documents such as government health policy documents, strategic plans, health financing policy documents, Universal Health Coverage policy documents, published literature, unpublished documents, media reports and National Health Accounts reports.

**Results**: The results show that each of the selected countries relies on a plurality of health insurance schemes with each targeting different groups. Additionally, many of the Social Health Insurance programs start by covering the formal sector first, with the hope of covering other groups in the informal sector at a later stage. Health insurance coverage for poor groups is very low, with targeting mechanisms to cover the poor in the form of exemptions and waivers achieving no desirable results.

**Conclusions**: The ability for Social Health Insurance programs to cover all groups has been limited in the selected countries. Hence, relying solely on social health insurance schemes to achieve Universal Health Coverage may not be plausible in Africa. Also, highly fragmented risk pools impede efforts to widen the insurance pools and promote cross-subsidies.

## Background

Universal Health Coverage (UHC) has become a political priority for many African countries but achieving this goal has been a challenge. It is estimated that globally around 400 million people lack access to at least one essential health service and that around 100 million are impoverished every year because of healthcare costs [1]. More than 40% of Total Health Expenditure (THE) in Low-Middle-Income countries (LMICs) come from out-of-pocket health expenditure [1]. In many Sub-Saharan African (SSA) countries, evidence shows that the poor bear the highest burden of diseases and experience high levels of catastrophic health expenditures [2].

To achieve UHC requires commitment to three key principles: mobilizing adequate resources to ensure coverage, providing quality care through strengthening the health service delivery system and ensuring that health services are accessible to all, especially poor and vulnerable individuals [3]. Since the 1980s, many African countries have instituted a number of reforms to improve the health sector. Notable amongst them, were the Structural Adjustment Programs (SAP), which was the response of the World Bank and the International Monetary Fund (IMF) to the African economic crisis of the 1970s. The SAP pushed for privatizing the public sector services, reducing subsidies and cutting public support for social services [4]. In many developing countries, the broad strategy of health reforms has been to reduce government spending on the health sector and curb the shortages of essential medicines and medical supplies through initiating copayment schemes [5]. However, increasing user fees without improving the quality of services provided resulted in reduced use of health facilities [6].

Contributory insurance and tax revenues are identified as the two main methods used to pool health system financing, and these differ by the way funds are pooled, either directly under the insurance approach or indirectly under the tax revenue approach [7]. Governments often charge user fees in an attempt to raise additional funds for the health system and restrict demand [8]. However, the World Health Organisation (WHO) recommends that total out-of-pocket payments (OOP) should not exceed 15–20% of national health expenditures to reduce the incidence of catastrophic health payments [9].

Health financing refers to the function of a health system concerned with the mobilisation, and allocation of money to cover the health needs of the people, individually and collectively, in the health system [10]. Many countries differ in how these functions are carried out and demonstrably, there is no unique model for health financing. The current trend in many countries in Africa is to adopt a mix of different approaches for health financing. These include combinations such as government tax financing for public health facilities through government budgets, social health insurance or voluntary health insurance, and other direct payments [11,12]. Also, most Social Health Insurance (SHI) schemes are changing and do not only rely solely on wage-based deductions. For example, Ghana’s National Health Insurance Scheme (NHIS) includes insurance premiums, payroll tax and earmarked value-added tax (which accounts for 72% of funding) into a single system with a common benefit package. Mexico imposes tax on sugary soft drinks to raise additional funds for its health sector [13].

Health financing strategies in LMICs in Africa vary broadly by geographic region and social context. A number of health reforms have been enacted across SSA countries to enable them to attain UHC. Also, the principal features of the health financing systems are influenced by the need to underscore the Sustainable Development Goals (SDGs) backed by the national development efforts in each country. The notable differences between each country in terms of their health financing strategies also show how health systems are influenced by social, cultural, economic and political factors. Consequently, these differences result in creating challenges that are context specific, suggesting that countries will continue to find the right policy solutions to solve these evolving problems [14]. Despite these differences, LMICs in Africa share similar health financing goals: universal coverage of services, financial protection, quality and efficiency.

Although many African countries have experienced reasonable economic growth in the past two decades, improvements in the health outcomes have been slow and uneven. As the disease burden transitions to a mix of communicable and non-communicable diseases, there is an increasing demand for health services and the need for more investments in health systems across the region. Recent epidemics such as Ebola and the COVID-19 have revealed the apparent weakness within some health sectors in low-income countries [15]. Accelerating progress towards UHC in Africa will involve measures to ensure equitable access to health services. Since SHI has been identified as a possible route, we need to consider how effective this system of health financing is with regard to achieving the key UHC goals.

The Global Health 2035 outlines an investment framework led by the Lancet Commission, endorsing the call for progressive ‘universalism.’ Here, the move towards UHC target must include poor and marginalised groups from the outset as opposed to health reforms that considers these groups at a much later stage [16]. The Global Health 2035 agenda proposes two progressive pathways towards UHC. First, to ensure that coverage is universal and secondly that all people (not only the poor) are assured of the same minimum set of benefits [17]. This makes the timing of this study even more appropriate.

## Defining social health insurance

Health insurance systems are either voluntary, where people (or firms on behalf of their employees) choose to join or remain uninsured; or compulsory, where people are compelled by law to join an insurance programme. Health financing through compulsory insurance contributions is often a challenge for countries with a large informal base where enforcement has been found to be difficult. Social health insurance schemes allow people to contribute to a health fund which guarantees them access to a specified package of health services, often legislated by the government [18]. Contributions tend to come from obligatory deductions from employees’ salaries or their employers. This is often on a sliding scale, with the richer people contributing more than poor people in absolute terms. These contributions are paid directly into a health insurance fund [18].

Theoretically, SHI relies on the pooling of health revenues mandatorily and in principle this should ensure an equitable distribution across different population groups. This system has proved effective in countries with a large formal sector such as Germany and the Netherlands. These pooling schemes ensure cross-subsidisation from the rich to the poor and from the healthy to the sick. Social health insurance schemes are therefore built on solidarity principles. Another justification for SHI is that there is more transparency in the way that funds are collected. The funds are usually collected by an independent body and therefore free from political influences [19]. The challenge in implementing SHI, however, is ensuring that the scheme remains mandatory for all citizens. This prevents situations where the pools are dominated by poorer and sicker individuals, thereby weakening the ability of the schemes to cross-subsidise [20].

While SHI can enable countries attain UHC, as seen in some high-income countries, the potential to replicate this system in LMICs and expect similar results have been less so [21]. It is important to note that high-income countries using SHI such as Germany have taken over 100 years to reach UHC, often in an incremental manner, rather than through a transformational change of structures already in existence [22,23].

The key contribution of this study to current knowledge lies in the number of policy interventions that will be considered to determine how to make health financing systems more equitable and sustainable. The study investigates whether SHI schemes are effectively and efficiently covering all groups in Ghana, Ethiopia, Kenya, Rwanda and Tanzania. The objective is to draw out the evidence on what the selected countries are doing in terms of expanding insurance coverage and ensuring coverage goals are met.

## Methods

We searched PubMed and Google Scholar for articles published between 2000 and 2016. Search terms included ‘health financing’ OR ‘social health insurance’ AND ‘Ghana’ AND ‘Kenya’ AND ‘Ethiopia’ AND ‘Rwanda’ OR ‘Tanzania’ OR ‘community health insurance’ OR ‘financing’ OR ‘strategy’ OR ‘poor.’ Further, articles were identified through a snowballing technique. We included only articles in English that discussed a combination of key concepts, including social health insurance, health financing strategies, insurance coverage, universal health coverage and implications of different health financing strategies on health insurance coverage. Finally, we supplemented the literature search with a search of grey literature using similar search terms, including health policy reports and national financing strategic response plans, UHC policy documents, published literature, unpublished documents, media reports and National Health Accounts (NHA) reports to analyse and to provide the challenges with the implementation of SHI schemes in the selected countries. The National Health Account is an internationally accepted tool used to estimate a country’s total spending on health activities [24]. NHA provides amongst others, information on the distribution of health spending from major financing sources.

Besides the desk review of key documents, the study included reflections from personal communication with key experts, advocates and government officials in some of the selected countries. The interviews gave an opportunity to understand the political underpinnings of UHC goals, pro-poor health financing strategies, current challenges with implementation and other relevant topics. Also, the results of the study were presented in a number of major health conferences in 2017 and 2018 which allowed the authors to receive further inputs from participants.

### Selection criteria

Countries were identified for inclusion in the study on the basis of the following criteria: (1) sub-Saharan LMICs; (2) countries that have introduced SHI programs; (3) countries with at least 10 million inhabitants. The study identified five countries that met the above criteria – Ghana, Rwanda, Tanzania, Kenya and Ethiopia.

## Results

### Key health outcome and economic indicators

Table 1 summarises key health outcome and economic indicators across the five countries. All five have high maternal mortality rates, but a much improved infant and under 5-years mortality ratios. Rwanda records the lowest of these ratios. There is wide variation in Gross Domestic Product (GDP) per capita, with Ghana having the highest GDP per capita, followed by Kenya.

**Table 1.** Country profiles of the five selected countries in the study

	Ghana	Kenya	Ethiopia	Tanzania	Rwanda
Total population (million)	30 (2019)	52 (2019)	112 (2019)	58 (2019)	12.6 (2019)
GDP per capita (current US$)	2,202 (2019)	1,816.5 (2019)	857.5 (2019)	1122.1 (2019)	801.7 (2019)
Gross Domestic Product Growth	6.5 (2019)	5.4 (2019)	8.3 (2019)	5.8 (2019)	9.4 (2019)
GNI per capita, Atlas method (Million US$)	0.67 (2019)	0.91 (2019)	0.95 (2019)	0.60 (2019)	0.10 (2019)
Inflation (%)	7.2 (2019)	4.7 (2019)	15.8 (2019)	3.5 (2019)	3.4 (2019)
Life Expectancy at Birth	64 (2018)	66 (2018)	66 (2018)	65 (2018)	69 (2018)
Infant Mortality (deaths per 1,000 live births)^a^	34 (2019)	32 (2019)	37 (2019)	36 (2019)	26 (2019)
Under 5 Mortality Rate (deaths per 1,000 live births)^6^	46 (2019)	43 (2019)	51 (2019)	50 (2019)	34 (2019)
Maternal Mortality Rate (Per 100,000) [UNICEF Database, 2017]	308	342	401	524	248
Out-of-pocket expenditure as % of THE in 2017	40	24	34.4	24	6.25

Source: http://data.worldbank.org/indicator/SH.XPD.OOPC.TO.ZS.

^a^http://dhsprogram.com/Where-We-Work/Country-List.cfm.

#### Strategies for financing health reforms

This section presents the various health financing strategies being implemented in each of the selected countries. From the literature reviewed, each of the countries relies on different types of health insurance schemes. These range from national health insurance schemes, community-based insurance schemes, private insurance schemes. Each of the aforementioned schemes either provides health coverage for specific segments of the population or the entire population.

Ghana, Tanzania, and Kenya have similar social health programs, although their target groups differ.Ghana’s National Health Insurance Scheme (NHIS) was enacted into law in 2003 and fully operationalized in 2005. The Scheme has UHC coverage as an underlying goal. This means that any Ghanaian is legally qualified to enroll in the scheme, although some segments of the population are exempt from payment of the premium, with exemption given to some segments of the population [25]. Tanzania implemented the National Health Insurance Fund (NHIF) in 1999 and offers compulsory coverage for all formal sector workers [26]. It also has a Community Health Fund (CHF) and *Tiba kwa Kadi* (TIKA) scheme that targets the informal sector [27]. Kenya’s National Hospital Insurance Fund (NHIF) started in the early 1960s [28]. The NHIF provides mandatory health coverage for all formal sector employees at calculated premiums on incomes as well as voluntary health coverage for its citizens in the informal sector at a premium [28]. Kenya also has community-based health insurance schemes (CBHIS) that target citizens in the informal sector and a Social Health Insurance Benefit (SHIB) that covers private-sector employees.

The informal sector comprises self-employment in small-unregistered enterprises and wage employment in unregulated and unprotected jobs and it is characterised by lower incomes. On the other hand, employees in the formal sector have contractual agreements with their employers and are expected to work for fixed hours and receive fixed salaries in addition to other benefits. For instance, the Social Security and National Insurance Trust (SSNIT) contributions from salaried workers in Ghana are levied at source and therefore it is much easier to collect such mandatory payments. However, SSNIT contributions account for only 20% of the NHIS funding in Ghana. Rwanda has in the past few years prioritised the health sector and is making progress towards UHC. The country’s community-based health insurance scheme (Mutuelles de santé) covers 84% of the population.

Ethiopia piloted a CBHIS program in 13 districts in 2010/2011, which is now being scaled up to 185 districts in four regions [29]. Rwanda also operates a CBHIS (*Mutuelles de Santé)*, which is an expanded program from a pilot phase introduced in 2003 [30,31]. Rwanda is the only country in SSA to have achieved enrollment rates in formal insurance schemes in excess of 80% of the population [30]. It is important to note that membership in Rwanda’s CBHIS is now mandatory which in effect has transformed it from a voluntary CBHIS programme to a compulsory (and heavily subsidized) SHI programme.

#### Out-of-pocket expenditure

The relative share of financing sources in each of the five countries comprises the following: donors, public sector, households (who pay insurance premiums and pay out-of-pocket to providers), and other private sector entities, such as private and parastatal enterprises. Out-of-pocket (OOP) payments is simply where people pay health service providers when they use their services. Although a simple method of health financing, the use of OOP payments as a means of financing health care raises a number of equity issues. Under this financing mechanism, health services are not allocated according to need but according to people’s ability to pay which negatively affects poor people. The risk of impoverishment is heightened if the poor have no other choice but to seek care [32].

Relying on OOP payments as a source of revenue for the health sector has been found to be regressive and also limits the use of healthcare services by vulnerable groups [33]. The level of OOP payments gives an indication of the level of financial protection in different countries. Globally, 170 million people are forced to spend more than 40% of their household income on medical treatment [34]. This is however higher than the recommended 15–20% which is the recommended level to ensure a low risk of catastrophic health spending [35]. A high percentage of OOP payments indicate limited financial protection. This implies that making efforts to reduce OOP payments will drive countries closer to achieving UHC. Comparatively, the selected countries in this study tend to have lower proportions of OOP payments than other African countries such as Nigeria or Sudan. Of the selected case studies, Ghana reports the highest level of OOP payments (40%) followed by Ethiopia at 34.4%. Rwanda has lowest level of OOP payments at 6.3%.

The structure of health financing has a large influence on the level of risk pooling. Ethiopia’s CBHIS relies mainly on premium payments, which account for 70% of its revenue [36]. In Rwanda, Kenya, Ethiopia and Tanzania, where CBHIS are attempting to cover the informal sector, contributions are levied at flat rates for households with the exception of Rwanda [27,28]. In Rwanda, a change in mutuelle policy in April 2010 introduced a sliding scale (based on ownership of assets) for premium payments to ensure a more equitable access to health services. With the Community Health Fund (CHF) in Tanzania, the government matches all contributions by members through a matching grant – matching grants are allocated based on the amount of premium revenue collected by each district or council [37]. Also, contributions to the Community Health Fund (CHF) are decided at the council level, and each household contributes the same amount regardless payment ability, giving them access to free health care at primary public health facilities [27,38,39].

In Ghana, by the end of 2013, 92% of the NHIS budget was financed through a 2.5% national health insurance levy on goods and services collected under the Value Added Tax (VAT), a and 2.5 percentage points of SSNIT contributions. About 3% of funding was from household premiums [25]. On the other hand, Rwanda’s CBHIS is predominantly funded by member premiums (66%), followed by the government and external funds (24%) which supports the operational costs of the schemes [32]. It is important to point out that the health services in Rwanda are heavily subsidised [40]. In Kenya, 34% of Current Health Expenditure (CHE) in 2012/2013 was mobilised through central government schemes, followed by household OOP payments (29%) and non-profit institutions serving households (NPISH) constituting 19% of Community Health expenditure (CHE) funds, Voluntary Health insurance schemes (9%), SHI schemes (5%), Enterprise Finance Schemes (3%) and others (1%). Clearly, in countries where risk pooling is high, we see less OOP as seen in the case of Rwanda.

Box 1 highlights the fragmented health financing system in Tanzania and efforts being made towards a national insurance scheme. Tanzania’s case study is unique in the fact that it shows that merging pools requires political agreement and demands capacity to manage pooled funds. The information gathered shows that attempts to merge separate schemes in Tanzania have been met with resistance.

**Table:** 

**Box 1: Tanzania’s fragmented health insurance system** Tanzania’s continued reliance on donor financing and low uptake of national health insurance reveals challenges in the country’s drive towards UHC. A major constraint is the large number of insurance schemes that serve different groups within the country listed below: National Health Insurance Fund (NHIF) Community Health Fund (CHF) Tiba Kwa Kadi (TIKA) Social Health Insurance Benefit (SHIB) Private insurance schemes Micro-insurance schemesThe formal sector is served by the NHIF, SHIB and private insurance companies. The informal sector relies on community based schemes such as the CHF, TIKA and micro-insurance schemes run by private sector firms. However, these schemes with the exception of the NHIF, are voluntary, have low penetration and limited financial and risk-pooling. Private micro-insurance schemes are few, and concentrated in small pockets of the country. There are efforts to build Public Private Partnerships (PPPs) to improve the CHF. For example, since 2011, PharmAccess has been working with the Kilimanjaro Native Cooperative Union (KNCU- made up of 68 primary societies) and the NHIF to establish and implement a health plan for the cooperative members and their dependents in the Kilimanjaro region. This is known as the ‘improved CHF’ (iCHF) and is supported with 50% of matching grant from the central Tanzania government like the other CHF schemes. However, the iCHF utilises the infrastructure of NHIF with technical expertise provided by PharmAccess.Efforts by the Tanzanian government to cross subsidize and widen the insurance pool has faced many oppositions in recent years. Recently, the NHIF, has been expanded to cover the self-employed and also allows voluntary membership outside the public sector. The NHIF has also been mandated by the Ministry of Health and Social Welfare (MoHSW) of Tanzania to support and manage the Community Health Fund (CHF) implementation in all districts of the Tanzania Mainland. This is to improve the operational functions of the CHF at the council level. The government has set up a ministerial task force comprising members from the MoHSW, NHIF, academic institutions and other relevant institutions to draft a policy for UHC. This policy will be the government’s blue print to improve health insurance coverage to all regions in the country.

## Discussion

Even though SHI is considered a key mechanism for providing financial protection, less well documented in the literature is the experience of how countries in Africa who have adopted this health financing strategy have tackled the issue of ensuring coverage across all populations of interest. Many of the SHI programs start by covering the formal sector first, with the hope of covering other groups in the informal sector at a much later stage. However, for countries to make progressive steps towards UHC, the investment should be in covering all groups right from the beginning [41]. From the experience of these selected countries, the use of SHI as an equitable financing mechanism is impeded by a large informal sector especially when membership to the health insurance scheme is voluntary.

The question is whether the level of health insurance coverage reflects on the goals of the SHI schemes, which is to cover all population groups such as the poor and other marginalised groups. In cases where the informal sector is not mandated to be part of the insurance scheme, this financing strategy fails, and such countries are left with a two or more tiered system with a strong resistance to merging them. An example is the case of Tanzania, where the country struggles to merge its two-tiered system after more than a decade of introducing SHI schemes [42]. Given the presence of large informal sectors characteristic of many LMICs, such schemes have been less successful, as mandatory membership has been difficult to enforce [43]. Increasingly, SHI schemes in developing countries have often been introduced first in the formal sector with the intention of a future rollout to the informal sector.

It still remains that moving towards greater reliance on public funding will bridge the gap between the poor and rich with regards to access to healthcare. The evidence suggests that health financing using predominantly government revenues have enabled some developing countries such as Brazil, Mexico and Thailand to make rapid progress towards universal coverage [43]. Thailand’s Universal Coverage Scheme (UCS) mostly financed through general government revenue since 2002, has reduced the average out-of-pocket health expenditure to 8%, and almost 100% health protection coverage had been reached by 2016 [44]. In 2003, the Mexican government reformed its public health sub-system to increase the financial protection of about 50 million people who were not covered by any of the existing social insurance schemes, leading to an improvement in access to healthcare services [45]. Thailand operates a system where the government bears themajority of its national health expenditure to ensure health insurance coverage for the entire population [46].

Arguably, administering and managing SHI programs is associated with notable challenges in the selected countries under review. Some of these key challenges include inequitable health insurance coverage, unsustainable financing of the schemes, highly fragmented risk pools, low enrolment into the schemes, limited coverage of the poor and vulnerable groups and ineffective exemption strategies.

Ultimately, inequitable health insurance coverage was observed in most of the selected countries. The goal of social health programs is to ensure that all citizens are protected against catastrophic healthcare expenditure. Although the schemes have demonstrated some successes in that regard, empirical evidence suggested that SHI programs in Africa are pro-rich, pro-educated, and pro-urban [47–49]. In Ghana, for instance, many studies emphasise the fact that individuals in high wealth quintiles were more likely to enrol onto the health insurance scheme than people in the lowest wealth quintile [47–49]. Dalinjong and Laar [50] note that non-enrolees only visited hospitals in times of critical ill-health due to the high cost of healthcare under the NHIS for non-enrolees. Additionally, women in the highest wealth quintile in the north of Ghana and with at least high school level education were more likely to be enrolled [51]. Similar observations were made in Ethiopia, where wealthier citizens were more likely to enrol onto the CBHIS [52].

Highly fragmented risk pools impede efforts to widen the insurance pools and promote cross-subsidies. Resistance to cross-subsidisation across risk pools means most insurance pools remain small and unsustainable in the long term as seen in Tanzania. A broad-based and equitable coverage as expected in traditional SHI programs has been found missing in the selected countries. For instance, Kenya and Tanzania have for the past decade struggled to gain consensus to establish National Health insurance schemes but with limited success [53–55].

There is evidence to show the limitation in relying on voluntary household contributions to sustain CBHIS. According to Ethiopian Health Insurance Agency report, the CBHIS observed a drop in both membership renewals and new registrations. In Fogera Woreda, one of the pilot districts, for example, it was reported that about 33% of all its CBHI members in the year 2013 were non-paying (subsidised by government funding) members and total membership declined by 23% from 2012 [36]. In such circumstances, where CBHIS are characterised by low enrolment, declining membership and low community contribution, the financial sustainability of a scheme is often jeopardised. Although it has been suggested that the sustainability of the scheme will depend on premiums from households in the community, this is more likely to be guaranteed by subsidies (public or external) as occurs in Rwanda and Tanzania.

Besides, the operational challenges, the wider concern is the sustainability of the SHI programs in the selected countries. The NHIS in Ghana is primarily tax-based with premium contributions accounting for just under 5% of revenues. The heavy reliance on taxes (92%) suggests that the financial security of NHIS depends largely on the country’s pace of economic growth [49]. The financial sustainability of the scheme is questionable in the long term without government budgetary support. Similarly, in Kenya, the government is unwilling to commit adequate budgetary resources to finance the health insurance schemes although it relies on the multiplicity of sources of finance [56]. The same can be said of the CBHIS in Ethiopia which relies on government and donor financing to support subsidies for the poor [57].

## Limitations

This study aims to provide information on the impact of the health financing reforms at the national level, in order to associate the policy process to relevant health system outcomes wherever possible. The study includes indicators of health expenditure, budget allocation in the public sector among others. There is an assessment of OOP expenditure and access to services in the selected countries. However, it is important to note that while these may be good indicators for measuring the extent of financial protection and coverage of health services, using these alone limits information on those who fail to access health care completely due to the cost of getting to the health facilities [35,58].

The study is also limited by the lack of data on some of the indicators. However, the study addresses the main objectives but remains cautious in generalising based on evidence from five country case studies. Relying on the information from interview data to triangulate the existing data is also limited by what respondents can remember.

## Conclusion

The study aimed to review evidence on the extent to which SHI covers all groups in countries where they have been introduced. The study has shown that SHI programs in SSA differ in structure and financing arrangement and the also in terms of population coverage, arrangement and proportion of cost covered through the contribution. In terms of population coverage, the results have shown that with the exception of Rwanda and Ghana, a very small proportion of the population is covered by health insurance. This situation is often worsened by high fragmentation of the risk pools and the voluntary nature of the schemes, undermining the potential for income cross-subsidisation.

The ability of the SHI schemes to offer adequate financial risk protection depends on the government’s commitment to design the schemes as part of a national financing strategy as has been demonstrated by Rwanda and Ghana. The evidence shows that majority of the schemes in SSA are not mandatory. Enforcing mandatory contributions has been one of the biggest challenges faced by Ghana’s NHIS. Rwanda has been successful in this regard, having been able to draw a highly fragmented scheme into a single pool but at the same time keeping the positive aspects of community-based scheme such as solidarity and community ownership of the schemes. Experiences in Uganda, China, Rwanda, and Thailand illustrate the critical importance of coordination across social health protection programmes, and of placing health insurance rationally into a broader social protection framework.

Whilst, no two countries have the same health financing strategy it has been possible to review how the combination of health financing strategies have drawn some countries further away from reaching their UHC targets. The evidence suggests that these equity goals cannot be achieved by a specific form of SHI scheme, whether based on a single-payer system or a multi-level payer one, or some combination of the two. Malawi which has no form of SHI has one of the lowest levels of OOP payments at 10.6% and the country with the highest proportion of government expenditure (11.4%).

Finally, we note the relevance of such multi-country studies like this one, which allow for some validation and knowledge building. The key conclusion is that fragmented risk pools, discourage income cross-subsidisation amongst the pools, making them unsustainable in the long term. We recommend that tax-funding arrangements should be provided to adequately cover all groups.

## Data Availability

Not applicable.

## Paper context

Many African countries share similar health financing goals: universal coverage of services, financial protection, quality and efficiency. However, health insurance coverage for poor groups is very low, with targeting mechanisms to cover the poor in the form of exemptions and waivers achieving no desirable results. Highly fragmented risk pools impede efforts to widen the insurance pools and promote cross-subsidies. Relying solely on social health insurance schemes to achieve Universal Health Coverage may not be plausible in these countries.

## References

[1] World Health Organization, World Bank. Tracking universal health coverage: first global monitoring report. 2015 Jun. Available from www.who.int/healthinfo/universal_health_coverage/report/2015/en

[2] Ezat Wan Puteh S, Almualm Y. Catastrophic health expenditure among developing countries. Health Syst Policy Res. 2017;4. DOI:10.21767/2254-9137.100069

[3] World Health Organization. Making fair choices on the path to universal health coverage: final report of the WHO Consultative Group on Equity and Universal Health Coverage. Geneva: World Health Organization; 2014. p. 1–9. 10.2471/BLT.14.139139 PMC4047814 24940009

[4] Helleiner GK. Accelerated development in sub-Saharan Africa, an agenda for action. J Dev Econ. 1983;13:259–264.

[5] World Development Report 1993. Investing in health. Oxford: The World Bank, Oxford University Press; 1993. No. of pages: 329. ISBN 0-19-520890-0.

[6] Sahn DE, Bernier R. Evidence from Africa on the intrasectoral allocation of social sector expenditures. Cornell Food and Nutrition Policy Program Working Paper No. 4.5; 1993.

[7] Kutzin J. Bismark meets Beveridge on the Silk Road: coordinating funding sources to create a universal health financing system in Kyrgyzstan. Bull World Health Organ. 2009;87:549–554. 19649370 10.2471/BLT.07.049544 PMC2704031

[8] Akin JS, Birdsall N, de Ferranti D. Financing health services in developing countries: an agenda for reform. A World Bank Policy Study. Washington: DCWorld Bank; 1987.

[9] Xu K, Evans DB, Kawabata K, et al. Household catastrophic health expenditure: a multicountry analysis’. Lancet. 2003;362:111–117. 12867110 10.1016/S0140-6736(03)13861-5

[10] The world health report 2000 – health systems: improving performance. Geneva; World Health Organization; 2000 [cited 2020 Oct 23]. Available from: (http://www.who.int/whr/2000

[11] Lagomarsino G, Garabrant A, Adyas A, et al. Moving towards universal health coverage: health insurance reforms in nine developing countries in Africa and Asia. Lancet. 2012;380:933–943. 22959390 10.1016/S0140-6736(12)61147-7

[12] Spaan E, Mathijssen J, Tromp N, et al. The impact of health insurance in Africa and Asia: a systematic review. Bull World Health Organ. 2012;90:685–692. 22984313 10.2471/BLT.12.102301 PMC3442382

[13] Boseley S. Mexico enacts soda tax in effort to combat world’s highest obesity rate [Internet]. The Guardian. 2020 [cited 2020 Apr 16]. Available from: https://www.theguardian.com/world/2014/jan/16/mexico-soda-tax-sugar-obesity-health

[14] Cotlear D, Nagpal S, Smith O, et al. Going universal: how 24 developing countries are implementing universal health coverage from the bottom up. Washington (DC): World Bank; 2015.

[15] Chia T, Oyeniran OI. Will Africa experience a spike in COVID-19 cases? Asian Pac J Trop Med. 2020;13:285–287.

[16] Preker AS, Carrin G. Health financing for poor people: resource mobilization and risk sharing. Vol. 434. Washington, DC: World Bank Publications; 2004.

[17] Millicent KA, Van der Geest S. Why are the poor less covered in Ghana’s national health insurance? A critical analysis of policy and practice. Int J Equity Health. 2016;15:34. 26911139 10.1186/s12939-016-0320-1 PMC4766646

[18] McIntyre D. A tale of two visions: the changing fortunes of social health insurance in South Africa. Health Policy Plan. 2003;18:47–58. 12582107 10.1093/heapol/18.1.47

[19] Schäfer W. Health systems in transition. Copenhagen: European Observatory on Health Systems and Policies; 2010.

[20] Gilson L, McIntyre D. Removing user fees for primary care in Africa: the need for careful action. BMJ. 2005;331:762–765. PMID: 16195296. 16195296 10.1136/bmj.331.7519.762 PMC1239983

[21] Hercot D, Meessen B, Ridde V, et al. Removing user fees for health services in low-income countries: a multi-country review framework for assessing the process of policy change. Health Policy Plan. 2011;26:ii5–ii15. 22027919 10.1093/heapol/czr063

[22] Bärnighausen T, Sauerborn R. One hundred and eighteen years of the German health insurance system: are there any lessons for middle- and low-income countries? Soc Sci Med. 2002;54:1559–1587. 12061488 10.1016/s0277-9536(01)00137-x

[23] Kutzin J. Experience with organizational and financing reform of the health sector. World Health Organization, Current Concerns SHS Paper No. 8. Geneva; 1995.

[24] World Health Organization. Guide to producing National Health Accounts. Geneva: WHO; 2003. Available from <www.who.int/nha/docs/English_PG.pdf>

[25] National Health Insurance Authority, annual Report (NHIA). Accra, Ghana. 2010; p: 16-21. [Internet]. Sciepub.com. 2020 [cited 2020 Apr 16]. Available from: http://www.sciepub.com/reference/38391

[26] Marwa CW. Provision of national health insurance fund services to its members; pain or gain? Unified J Sport Health Sci. 2016;2:1–6.

[27] Mills A, Ataguba J, Akazili J, et al. Equity in financing and use of health care in Ghana, South Africa, and Tanzania: implications for paths to universal coverage. Lancet. 2012;380:126–133. 22591542 10.1016/S0140-6736(12)60357-2

[28] Deolitte CL. A strategic review of NHIF and market assessment of private prepaid health schemes. Nairobi: Ministry of Medical Services; 2011.

[29] Feleke S, Workie M, Hailu Z, et al. Ethiopia’s community-based health insurance: a step on the road to universal health coverage. Washington (DC): USAID; 2015.

[30] Makaka A, Breen S, Binagwaho A. Universal health coverage in Rwanda: a report of innovations to increase enrolment in community-based health insurance. Lancet. 2012;380:S7.

[31] Kunda T. Increasing equity among community based health insurance members in Rwanda through a socioeconomic stratification process. Paper presented at the Third International Conference of the African Health Economics and Policy Association, Nairobi, Kenya. 2014.

[32] Kalisa I, Musange S, Collins D, et al. The development of community-based health insurance in Rwanda - Experiences and lessons. Medford (MA, USA): University of Rwanda College of Medicine and Health Sciences - School of Public Health, Kigali, Rwanda and Management Sciences for Health; 2015 July.

[33] Wagstaff A. Social health insurance re-examined. Health Econ. 2010;19:503–517. 19399789 10.1002/hec.1492

[34] ILO. Social health protection: an ILO strategy towards universal access to health care. Geneva: International Labour Organization. [Google Scholar]; 2008.

[35] Boerma T, Eozenou P, Evans D, et al. Monitoring progress towards universal health coverage at country and global levels. PLoS Med. 2014;11:e1001731. 25243899 10.1371/journal.pmed.1001731 PMC4171369

[36] EHIA. Evaluation of community-based health insurance pilot schemes in Ethiopia: final report. Addis Ababa: Ethiopia Health Insurance Agency; 2015.

[37] Macha J, Harris B, Garshong B, et al. Factors influencing the burden of health care financing and the distribution of health care benefits in Ghana, Tanzania and South Africa. Health Policy Plan. 2012;27:i46–i54. 22388500 10.1093/heapol/czs024

[38] Humba E. Pioneering social health insurance in Tanzania: the case of the National Health Insurance Fund (NHIF). Paper presented at the Proceedings of Swiss TPH Spring Symposium on Improving Access Through Effective Financing, Basel, Switzerland. 2011.

[39] Mtei G, Makawia S, Ally M, et al. Who pays and who benefits from health care? An assessment of equity in health care financing and benefit distribution in Tanzania. Health Policy Plan. 2012;27:i23–i34. 22388497 10.1093/heapol/czs018

[40] Nyandekwe M, Nzayirambaho M, Kakoma JB. Universal health coverage in Rwanda: dream or reality. Pan Afr Med J. 2014;17. DOI:10.11604/pamj.2014.17.232.3471 PMC4145275 25170376

[41] Jowett M, Brunal MP, Flores G, et al. Spending targets for health: no magic number. Health Financing Working Paper No. 1. Geneva: World Health Organization; 2016. Available from: http://www.who.int/health_financing/documents/no-magic-number/en/

[42] Borghi J, Maluka S, Kuwawenaruwa A, et al. Promoting universal financial protection: a case study of new management of community health insurance in Tanzania. Health Res Policy Syst. 2013;11:21. 23763711 10.1186/1478-4505-11-21 PMC3686629

[43] Acharya A, Vellakkal S, Taylor F, et al. Impact of national health insurance for the poor and the informal sector in low- and middle-income countries: a systematic review. London: EPPI-Centre, Social Science Research Unit, Institute of Education, University of London; 2012.

[44] Glassman A, Temin M Thailand’s universal coverage scheme. Millions saved: new cases of proven success in global health. Center for Global Development; 2016. Available from: http://millionssaved.cgdev.org/case-studies/thailands-universal-coverage-scheme

[45] García-Díaz R, Sosa-Rubí SG, Serván-Mori E, et al. Welfare effects of health insurance in Mexico: the case of Seguro Popular de Salud. PLoS One. 2018;13:e0199876. 29965976 10.1371/journal.pone.0199876 PMC6028097

[46] Tangcharoensathien V, Witthayapipopsakul W, Panichkriangkrai W, et al. Health systems development in Thailand: a solid platform for successful implementation of universal health coverage. Lancet. 2018 Mar 24;391:1205–1223. PMID: 29397200. 29397200 10.1016/S0140-6736(18)30198-3

[47] Jehu-Appiah C, Aryeetey G, Spaan E, et al. Equity aspects of the national health insurance scheme in Ghana: who is enrolling, who is not and why? Soc Sci Med. 2011;72:157–165. 21145152 10.1016/j.socscimed.2010.10.025

[48] Odeyemi I, Nixon J. Assessing equity in health care through the national health insurance schemes of Nigeria and Ghana: a review-based comparative analysis. Int J Equity Health. 2013;12:1. 23339606 10.1186/1475-9276-12-9 PMC3626627

[49] Witter S, Garshong B. Something old or something new? Social health insurance in Ghana. BMC Int Health Hum Rights. 2009;9:1. 19715583 10.1186/1472-698X-9-20 PMC2739838

[50] Dalinjong PA, Laar AS. The national health insurance scheme: perceptions and experiences of health care providers and clients in two districts of Ghana. Health Econ Rev. 2012;2:1. 22828034 10.1186/2191-1991-2-13 PMC3505458

[51] Akazili J, Welaga P, Bawah A, et al. Is Ghana’s pro-poor health insurance scheme really for the poor? Evidence from Northern Ghana. BMC Health Serv Res. 2014;14:1. 25494816 10.1186/s12913-014-0637-7 PMC4268792

[52] Mebratie AD, Sparrow R, Yilma Z, et al. Impact of Ethiopian pilot community-based health insurance scheme on health-care utilisation: a household panel data analysis. Lancet. 2013;381:S92.

[53] Chuma J, Okungu V. Viewing the Kenyan health system through an equity lens: implications for universal coverage. Int J Equity Health. 2011;10:22. 21612669 10.1186/1475-9276-10-22 PMC3129586

[54] Mulupi S, Kirigia 1 D, Chuma J. Community perceptions of health insurance and their preferred design features: implications for the design of universal health coverage reforms in Kenya. BMC Health Serv Res. 2013;13:474. 24219335 10.1186/1472-6963-13-474 PMC3842821

[55] Abuya T, Thomas M, Chuma J. Historical account of the national health insurance formulation in Kenya: experiences from the past decade. BMC Health Serv Res. 2015;15:1–11. 25884159 10.1186/s12913-015-0692-8 PMC4332452

[56] Karanja JW. Challenges in provision of universal health care by the National Hospital Insurance Fund, Kenya [Master’s]. Nairobi: University of Nairobi; 2014.

[57] Alebachew A, Hatt L, Kukla M, Nakhimovsky S. January 2014. Universal Health Coverage Measurement in a Low--Income Context: An Ethiopian Case Study. Bethesda, MD: HealthFinance & Governance Project, Abt Associates Inc.

[58] World Bank/World Health Organization. Monitoring progress towards universal health coverage at country and global level. Framework, measures and targets. Geneva: WHO; 2014. Available from: http://apps.who.int/iris/bitstream/10665/112824/1/WHO_HIS_HIA_14.1_eng.pdf 10.1371/journal.pmed.1001731 PMC4171369 25243899

